# Prevalence and force of *Plasmodium vivax* blood-stage infection and associated clinical malaria burden in the Brazilian Amazon

**DOI:** 10.1590/0074-02760210330

**Published:** 2022-06-24

**Authors:** Wuelton Monteiro, Stephan Karl, Andrea Kuehn, Anne Almeida, Michael White, Sheila Vitor-Silva, Gisely Melo, Jose Diego Brito-Sousa, Djane Clarys Baia-da-Silva, Alexandre Vilhena Silva-Neto, Vanderson Sampaio, Quique Bassat, Ingrid Felger, Ivo Mueller, Marcus Lacerda

**Affiliations:** 1 Fundação de Medicina Tropical Dr Heitor Vieira Dourado, Manaus, AM, Brasil; 2 Universidade do Estado do Amazonas, Manaus, AM, Brasil; 3 Walter and Eliza Hall Institute of Medical Research, Victoria, Australia; 4 Papua New Guinea Institute of Medical Research, Vector-Borne Diseases Unit, Madang, Papua New Guinea; 5 Melbourne University, Department of Medical Biology, Victoria, Australia; 6 Universitat de Barcelona, Barcelona Institute for Global Health, Hospital Clínic, Barcelona, Spain; 7 Institut Pasteur, Department of Parasites and Insect Vectors, Malaria: Parasites and Hosts, Paris, France; 8 Centro de Investigação em Saúde de Manhiça, Maputo, Mozambique; 9 Catalan Institution for Research and Advanced Studies, Pg Lluís Companys, Barcelona, Spain; 10 University of Barcelona, Hospital Sant Joan Déu, Pediatric Infectious Diseases Unit, Pediatrics Department, Barcelona, Spain; 11 Swiss Tropical and Public Health Institute, Basel, Switzerland; 12 University of Basel, Basel, Switzerland; 13 Fundação Oswaldo Cruz-Fiocruz, Instituto Leônidas & Maria Deane, Manaus, AM, Brasil

**Keywords:** molecular force of blood-stage infection, Plasmodium vivax, malaria transmission

## Abstract

**BACKGROUND:**

Understanding the epidemiology of malaria through the molecular force of the blood-stage infection of *Plasmodium vivax* (molFOB) may provide a detailed assessment of malaria transmission.

**OBJECTIVES:**

In this study, we investigated risk factors and spatial-temporal patterns of incidence of *Plasmodium* infection and clinical malaria episodes in three peri-urban communities of Manaus, Western Brazilian Amazon.

**METHODS:**

Monthly samples were collected in a cohort of 1,274 individuals between April 2013 and March 2014. DNA samples were subject to *Plasmodium* species*.* molFOB was calculated by counting the number of genotypes observed on each visit, which had not been present in the preceding two visits and adjusting these counts by the respective times-at-risk.

**FINDINGS:**

Respectively, 77.8% and 97.2% of the population remained free of *P. vivax* and *P. falciparum* infection. Expected heterozygosity for *P. vivax* was 0.69 for MSP1_F3 and 0.86 for MS2. Multiplicity of infection in *P. vivax* was close to the value of 1. The season was associated with *P. vivax* positivity [adjusted hazard ratio (aHR) 2.6 (1.9-5.7)] and clinical disease [aHR 10.6 (2.4-47.2)]. *P. falciparum* infection was associated with previous malarial episodes [HR 9.7 (4.5-20.9)]. Subjects who reported possession of a bed net [incidence rate ratio (IRR) 1.6 (1.2-2.2)] or previous malaria episodes [IRR 3.0 (2.0-4.5)] were found to have significantly higher *P. vivax* molFOB.

**MAIN CONCLUSIONS:**

Overall, *P. vivax* infection prevailed in the area and infections were mostly observed as monoclonal. Previous malaria episodes were associated with significantly higher *P. vivax* molFOB.

The global burden imposed by malaria remains high, with an estimated 241,000,000 cases and 627,000 related deaths in 2020.^1^ Although malaria remains endemic in 85 countries, most of the burden (95%) is confined to the African continent where *Plasmodium falciparum* predominates.^1^ In the Americas, *Plasmodium vivax* is the predominant species and caused over two thirds (68%) of all cases in 2020. Despite most endemic countries in Latin America have achieved a reduction of incidence since 2010, there were still more than half a million (653,300) cases in 2020, with Venezuela and Brazil accounting for more than half of these cases (65%).^1^ Between 2005 and 2019, Brazil achieved a 70% reduction of malaria cases, with 99% of all cases occurring in the Amazon region.^1^ ^,^ ^2^ ^,^ ^3^ Of special vulnerability are malaria-naïve individuals recently arrived from malaria-free areas and engaged in agricultural and forest-related activities such as logging, fishing, and mining.^4^ ^,^ ^5^ ^,^ ^6^ ^,^ ^7^ In the 1970s, increased industrial development demanded a large labor force, which evoked a large migratory influx to the peripheries of bigger cities such as Manaus. Such largely uncontrolled settlements have led to a gradual increase of malaria transmission in peri-urban areas.^3^

As in other regions, recent progress in malaria control in Brazil has been accompanied by an increasing proportion of cases caused by *P. vivax*, underscoring the challenges, namely relapses, yet to be addressed in control and management of this species.^8^ Understanding the epidemiology of relapses after primary infection is challenging, and an estimated 14-40% of individuals have detectable recurrences, even after treatment with primaquine.^7^ ^,^ ^9^ ^,^ ^10^ ^,^ ^11^ ^,^ ^12^ A second challenge is the early production of *P. vivax* gametocytes, along with asymptomatic cases, which enables the transmission of parasites even before the infected individual develops clinical symptoms. Thirdly, *P. vivax* parasite densities are generally lower than those seen in *P. falciparum* infections, especially in hypoendemic areas such as those in Brazil, and therefore require more sensitive diagnostic tools.^13^ Research has shown that a high proportion of submicroscopic and asymptomatic *P. vivax* infections usually exist in such settings, and that these infections can easily be missed by routine active and passive case detection.^13^ It has been shown that asymptomatically infected individuals are able to infect mosquitoes of the Amazon region and, therefore, these infections might fuel residual malaria transmission, thereby complicating malaria elimination.^14^ ^,^ ^15^ ^,^ ^16^ ^,^ ^17^

In view of these challenges to the elimination of malaria, it is important to gather data regarding spatial and temporal patterns of asymptomatic *Plasmodium* infections as well data on gametocyte carriage from low-transmission settings. Therefore, in this study, we investigated the risk factors and spatial-temporal patterns of the incidence of *Plasmodium* infection and clinical malaria episodes in a peri-urban area of Manaus, Western Brazilian Amazon. We measured the prevalence of blood-stage *P. falciparum* and *P. vivax* infections using polymerase chain reaction (PCR), the incidence of clinical cases, and estimated the molecular force of *P. vivax* blood-stage infection (molFOB) derived from molecular detection and genotyping of infections. molFOB is the observed number of new blood-stage infections, as identified by individual *Plasmodium* spp. genotypes, divided by the time-at-risk (i.e., the incidence of new blood-stage infections). molFOB is an important parameter for estimating exposure at the individual level and provides a detailed assessment of malaria transmission by measuring individual exposure and its burden.^18^ ^,^ ^19^ This information is important for malaria control and elimination measures.

## SUBJECTS AND METHODS

*Ethics statement* - This study was approved by the Brazilian National Ethics Committee (CONEP) (349.211/2013) and the Ethics Committee for Clinical Investigation of the Barcelona Hospital Clinic (7306/2012). All participants were informed about the objectives of the study, as well as the potential risks of and benefits from their participation in the study. An informed consent form was signed by all study participants or by a parent or legal guardian in case of participants that were under 18 years of age. Children between 12 and 17 years of age signed an additional assent form. Patients were treated according to the protocols of the Brazilian Ministry of Health.

**Fig. 1:**
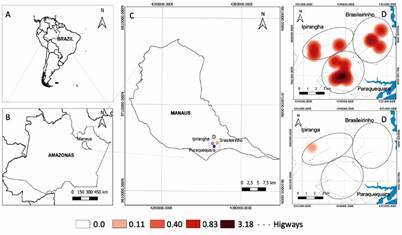
spatial representation of clinical *Plasmodium vivax* and *Plasmodium falciparum* cases. (A) South America; (B) Amazonas state; (C) Manaus municipality and study; (D) quantitative polymerase chain reaction (qPCR) detected *P. vivax*; (E) *P. falciparum* infections. Data are shown as incidence (cases/detections per person per year, aggregated to the household level). Increased diameter of the circles represents increased incidence. Maps were created using QGIS 2.18, with geodata collected for this study.

*Study design and subjects* - This cohort study was conducted between April 2013 and March 2014 in the Brasileirinho, Ipiranga and Puraquequara communities, which are located in the peri-urban area of Manaus (Fig. 1A). The rainfall and season data were obtained from the Brazilian National Institute of Meteorology (https://portal.inmet.gov.br/). The areas studied are endemic for malaria and have a high annual parasite index (IPA), despite the decrease in the IPA (Supplementary data - Figure). A detailed description of the study area has been presented elsewhere.^13^ In these areas, the population’s subsistence activities are concentrated in the agricultural sector and extractivism. Many people, however, work in the city of Manaus and, for this, they travel daily to the municipal seat. It is noteworthy that in this location there are several farms used for leisure and as religious retreats, to which many people from Manaus travel, especially on weekends and holidays. The occurrence of malaria in this population is common. According to a census performed by the Fundação de Medicina Tropical Dr Heitor Vieira Dourado’s (FMT-HVD) field team, before the start of the study, in 2012, the population of the study area was estimated to be approximately 2,400 inhabitants. Each community has access to a malaria clinic for microscopy-based malaria diagnosis and treatment.

All of the houses in the communities received visits. In the case of participants present at the residence, these were invited to participate in the study and be part of our sample. A total of 1,274 participants of any age were enrolled into the study.

*Sample collection* - For each study participant, a questionnaire was completed containing personal information such as age, gender, occupation, pregnancy, history of travel (displacement to Manaus, other cities and communities), as well as information on malaria preventive measures, previous malaria episodes and current health status. Upon enrolment and during monthly follow-ups, finger-prick blood samples (~300 μL) were collected using Microtainer^®^ tubes containing EDTA and sodium fluoride (Becton Dickinson, USA). In infants, blood was obtained by either heel or toe puncture. Within one hour of collection, 50 μL of blood were transferred into a reaction tube containing 250 μL of RNAprotect (Qiagen, Germany) in order to preserve RNA for downstream analyses, and 200 μL of whole blood was transferred to another reaction tube and was separated into a red blood cell (RBC) pellet and plasma.^20^

*Clinical symptoms* - In the case of symptoms related to malaria, or in the case of increased body temperature (> 37.5ºC), a thick blood smear (TBS) was prepared according to World Health Organization’s guidelines.^21^ When positive for malaria via a TBS, appropriate treatment was provided in accordance with the national guidelines of the Brazilian Ministry of Health.^22^ An asymptomatic infection was defined as presence of a malarial infection by TBS or qMAL PCR, but absence of fever and any other malaria related symptoms (chills, sweating, headache, vomit, and/or abdominal pain) at the moment of sample collection, or anytime in the preceding 48 hours. The submicroscopic infection was defined as detectable only by qMAL PCR and may be asymptomatic or not.

*Plasmodium spp. infection, clones and gametocyte carriage* - Pelleted RBCs, obtained from 200 μL of whole blood, were re-suspended in phosphate-buffered saline (PBS), and genomic DNA was extracted using a FavorPrep 96-well Genomic DNA kit (Favorgen, Taiwan), according to the manufacturer’s instructions. DNA was eluted with 2 x 100 μL of elution buffer and stored at -20ºC until assayed using PCR. RNA from 50 μL of whole blood, stored in RNAprotect, was extracted using an RNeasy Plus 96-well kit (Qiagen, Germany) and eluted in 50 μL of RNase-free dH_2_O, as described previously.^20^

All DNA samples were subjected to a generic *Plasmodium* species (QMAL) quantitative polymerase chain reaction (qPCR), which targeted a conserved region of the 18S rRNA gene.^20^ QMAL-positive samples were further analysed using species-specific qPCR assays that targeted the18S rRNA genes of *P. falciparum* and *P. vivax*, as previously described.^20^ ^,^ ^23^ For detection of *P. falciparum*, a modified reverse primer was used.^24^ For quantification of 18S rRNA gene copy numbers, in each experiment, three dilutions of control plasmids containing the respective amplicons were included in triplicates (10^2^, 10^4^ and 10^6^ copies/μL). For the genotyping of individual *P. vivax* clones, the molecular markers MSP1F3 and MS2 were typed using capillary electrophoresis for highly precise fragment sizing, which allowed for longitudinal follow up of individual parasite clones. Details of the genotyping methods have been described previously.^25^

Reverse transcription real-time polymerase chain reaction (RT-qPCR) assays were performed on RNAs from all *P. vivax* and/or *P. falciparum* positive samples to detect gametocyte-specific transcripts of the pvs25 (*P. vivax*) and pfs25 (*P. falciparum*) genes. For quantification of pvs25 and pfs25 transcript numbers, control plasmids containing the amplified region were included as standards in each run. All qPCR and RT-qPCR assays were performed on a real-time PCR System (7500 Fast, Applied Biosystems).

*Statistical analysis* - Data from questionnaires were imported into databases using the Cardiff TeleForm version 10.4.1 (Cardiff Software). Individual databases were combined in Microsoft Access 2010. For incidence calculations (molFOB and clinical malaria incidence), data from the subjects were censored on the last visit before two consecutively missed scheduled follow-up visits in order to reduce bias.^26^ Differences in proportions were tested for statistical significance using the McNemar X^2^ test with continuity correction. To achieve normal distribution, qPCR densities were expressed as log10-transformed 18S rRNA genomic copies/μL blood for asexual parasites, and log10-transformed pfs25 or pvs25 transcripts/μL blood for gametocytes. Geometric means of densities were calculated. Differences in densities of asexual or sexual-stage parasites were tested for statistical significance using Welch’s two-sample t-test.

The molFOB was calculated by counting the number of genotypes observed on each visit that had not been present in the preceding two visits (0-0-1 patterns) and then adjusting these counts by the respective times-at-risk. molFOB for *P. vivax* was determined for both genetic markers combined. Negative binomial regression models were used to assess the influence of different risk-factors on the incidence of *P. vivax* and *P. falciparum* gametocyte positivity as previously described, using positivity counts and times-at-risk over the entire period of observation.^26^ Since molFOB is a count variable that is measured per individual over a specific exposure time (time at risk) and is overdispersed, a negative binomial regression model was chosen in which the time at risk of exposure is used as an offset. If we define μj as the log of the number of genotypes at visit j, then for each infection pattern j (0-0-0 or 0-0-1) we have μj = exp (βxj + offsetj + νj), where β is a vector of regression coefficients, offset = log(exposure time) and νj follows a gamma distribution (to give a negative binomial distribution).

Incidence rate ratios (IRR) and adjusted IRR (aIRR) were calculated with their respective 95% confidence intervals. Because using the collapsed data to model molFOB for everyone does not allow for the analysis of time-changing covariates, factors influencing frequency of parasite positivity and frequency of clinical episodes within the study period were explored using multiple failure time models that allowed for time-changing covariates.^27^ For multiple failure time models, hazard rate ratios (HRR) were calculated with 95% confidence intervals. In these models, parasite positivity and clinical episodes were equivalent to a ‘failed’ outcome, respectively. In addition to the adjusted statistical models presented in the main manuscript, univariate analyses and multivariate analyses with backward selection are provided as Supplementary data. Versions of each model’s analysis were implemented with backwards-selection to eliminate non-significant covariates and resulted in the most parsimonious models. Statistical analyses were conducted using R v3.1.1 or STATA v14.

## RESULTS

*Study population* - In all, 1,274 individuals were enrolled at the beginning of the study. A total of 51.1% (651) were males and approximately half were aged between 18 and 59 years old (634; 49.7%). Nearly 40% (504) of the subjects claimed to have had more than three malaria episodes during their lifetime. A total of 17 individuals (1.3%) had experienced a malaria infection in the preceding two weeks and 40 (3.2%) had taken antimalarial drugs in the past two months. A total of 1,201 subjects (94.9%) had resided in the study area for more than two months. The number of individuals enrolled was similar in the three communities, Brasileirinho (n = 430), Ipiranga (n = 416) and Puraquequara (n = 428). The characteristics of the study population are summarised in Table I.

**TABLE I:** Baseline characteristics of the population enrolled in the study

Variable	Nº of participants N = 1,274	%
Gender		
Male	651	51.1
Age group (years)		
< 10	368	28.9
10-20	634	49.6
21-60	128	10.0
≥ 61	160	12.5
Occupation		
Agriculture	179	14.1
Office worker	170	13.3
House wife	218	17.1
Pre-school children	175	13.7
School children	331	26.0
Retired	63	5.0
Unemployed/Other	138	10.8
Previous infection (number) in the last two months		
1-3	364	28.6
> 3	504	39.6
Infection in past two weeks	17	1.3
Antimalarial in past two months	40	3.2
More than two months of residency	1,201	94.9
Community		
Ipiranga	430	33.8
Brasileirinho	416	32.7
Puraquequara	428	33.6

**Fig. 2:**
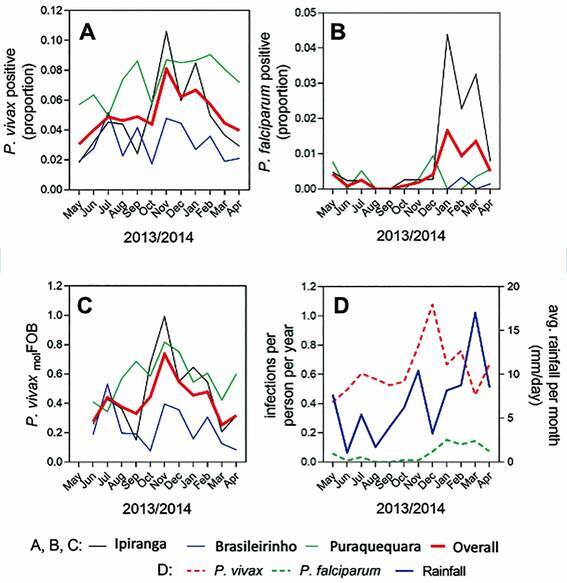
infection prevalence, molecular force of *Plasmodium vivax* infection and rainfall within the three communities. (A) prevalence of *P. vivax* infection in the three communities, and in in the entire study population; (B) prevalence of *Plasmodium falciparum* infection in the three communities, and in in the entire study population; (C) molecular force of *P. vivax* blood stage infection (molFOB) in the three communities and in the entire population; (D) rainfall and incidence of *P. vivax* and *P. falciparum* infections detected by polymerase chain reaction (PCR) in the entire study area.

*Prevalence by active case detection* - Monthly *P. vivax* prevalence by qPCR ranged from 2.5% in June 2013 to 6.5% in November 2013 (Fig. 2A). *P. falciparum* was not detected in August or September 2013, and the highest prevalence (~1.0%) occurred in March 2014 (Fig. 2B). Both species presented a similar seasonality profile, with a higher prevalence of *P. vivax* from October to February, and of *P. falciparum* from November to March, which coincides with the rainy season (Fig. 2C-D). The frequency of *P. vivax* asymptomatic cases was 67.3% (104/156); of asymptomatic was 65.6% (42/64), and 54.5% (12/22) of symptomatic *P. vivax* infections were submicroscopic. A total of 75.0 % (12/16) of the *P. falciparum* infections were asymptomatic, and four (35.7%) asymptomatic *P. falciparum* infections were submicroscopic. The geographic means of *P. vivax* and *P. falciparum* parasitemia in symptomatic participants were 258.07 and 372.76 parasites per mm^3^, respectively. *P. vivax* asymptomatic infections predominated in the Puraquequara community, although clinical cases were mostly seen in Ipiranga. For *P. falciparum*, asymptomatic infections and clinical cases were both predominant in the Ipiranga community. In the rainy season, fifty percent (43/86) of *P. vivax* infected individuals (qPCR) carried *P. vivax* gametocytes. In the dry season, 3.4% (70/2073) of participants had qPCR-detectable *P. vivax* infections, of which 42.9% (30/70) of infections were gametocyte positive.

*Risk factors associated with P. vivax positivity* - In the multivariate analysis shown in Table II, *P. vivax* infection was associated with the rainy season (Dec-May) [(HR = 2.56 (1.89-5.74), p < 0.001)], and community of residence (p = 0.002), with individuals living in Puraquequara having an aHR = 2.4 (1.1-2.3), when compared to Ipiranga. Age was associated with *P. vivax* infection (p = 0.001), with individuals aged 20-60 years having an aHR = 2.2 (1.1-2.5), when compared to children under 10 years. Bed net usage was significantly associated with higher *P. vivax* positivity [aHR = 1.06 (1.01-1.08), p = 0.005]. Indoor residual spraying (IRS) in the house was associated with lower risk of *P. vivax* infection [Table II and Supplementary data (Table I)].

**TABLE II:** Risk factors associated with *Plasmodium vivax* positivity, *P. vivax* clinical disease, and *Plasmodium falciparum* positivity. Adjusted hazards ratio (aHR) was calculated using a multiple failure time model

Risk factor	*P. vivax* positivity		*P. vivax* clinical disease		*P. falciparum* positivity	
	aHR	p	aHR	p	aHR	p
Community (ref. Ipiranga)						
Season (Ref: Jun-Nov)	2.56 (1.89-5.74)	< 0.001	10.56 (2.36-47.19)	0.002	0.08 (0.01-0.51)	0.008
Brasileirinho	1.01 (0.4-1.08)	0.002	0.11 (0.05-0.23)	< 0.001	0.04 (0.01-0.26)	< 0.001
Puraquequara	2.39 (1.11-2.31)		0.26 (0.14-0.5)		0.23 (0.07-0.71)	
Age group (ref. 1-10)						
10-20	1.36 (0.78-1.95)	0.001	1.01 (0.54-1.88)	0.02	0.78 (0.19-3.27)	0.300
21-60	2.22 (1.1-2.45)		0.63 (0.36-1.13)		1.33 (0.39-4.5)	
≥ 61	1.9 (0.71-2.15)		0.15 (0.04-0.63)		2.15 (0.47-9.94)	
Employed in agriculture^*a*^	0.82 (0.52-1.12)	0.162	0.43 (0.25-0.74)	0.002	1.48 (0.41-5.29)	0.546
Male	1.21 (0.91-1.62)	0.187	1.16 (0.77-1.75)	0.482	1 (0.4-2.47)	0.996
Bednet usage^*b,c*^	1.06 (1.01-1.08)	0.005	1.06 (1.01-1.11)	0.018	0.95 (0.85-1.06)	0.322
Travel frequency^2^	0.99 (0.99-1.01)	0.516	1.01 (1-1.01)	0.02	1 (0.95-1.05)	0.934
House treated with IRS^2^	0.96 (0.92-0.99)	0.022	0.92 (0.86-0.98)	0.008	1.08 (0.94-1.23)	0.278
Windows protected by screen^*a*^	0.78 (0.72-1.37)	0.958	1.56 (0.87-2.8)	0.135	1.05 (0.27-4.08)	0.946
Reported previous malaria	1.11 (0.56-1.58)	0.814	0.66 (0.17-2.64)	0.558	9.65 (4.45-20.92)	< 0.001

*a*: status at enrolment; *b*: as time-changing covariate (average observed at time of outcome); *c*: average bednet usage was defined as the proportion of times a person had answered `yes’ to the question: `Did you sleep under a bednet last night’ during ACD; IRS: indoor residual spraying.

*Risk factors associated with P. vivax clinical disease* - *Plasmodium vivax* clinical disease was associated with the rainy season [(aHR: 10.6 (2.4-47.2), p = 0.002)] (Table II). Living in the Brasileirinho [(aHR: 0.11 (0.05-0.23), p < 0.001)] and Puraquequara [(aHR: 0.26 (0.14-0.50), p < 0.001)] communities, working in agriculture [(aHR: 0.43 (0.25-0.74), p = 0.002)], and living in a house treated with IRS [(aHR: 0.92 (0.86-0.98), p = 0.008)] were all variables associated with a reduced risk of clinical malaria disease [Supplementary data (Table II)].

*Risk factors associated with P. falciparum positivity* - *Plasmodium falciparum* infection was associated with living in a community (p < 0.001), with individuals living in Brasilerinho [aHR = 0.04 (0.01-0.26)] and Puraquequara [aHR = 0.23 (0.07-0.71)] having lower positivity than individuals in Ipiranga. Individuals with previous malarial episodes had significantly higher *P. falciparum* positivity [(aHR: 9.7 (4.5-20.9), p < 0.001)] [Supplementary data (Table III)].

**Fig. 3:**
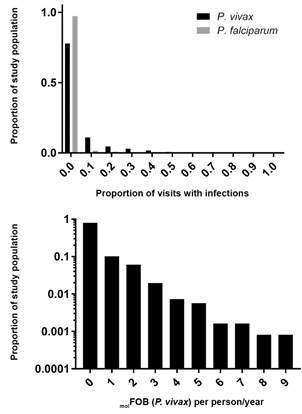
distribution of *Plasmodium* infections in the study population. (A) heterogeneity in the incidence of malaria infections; (B) *P. vivax* molFOB over the entire year of follow-up.

*Genetic diversity and multiplicity of infections* - The heterogeneity in the incidence of malaria infections is shown in Fig. 3A. Both *P. vivax* and *P. falciparum* infections were restricted to a small proportion of the study population. Overall, 77.8% of the population remained free of *P. vivax* infection and 97.2% of the population remained free of *P. falciparum* infection over the course of the study period. Based on the two markers (MSP1 F3 and MS2), expected heterozygosity for *P. vivax* was 0.69 (MSP1_F3) and 0.86 (MS2). Overall, the multiplicity of infection was close to the value of 1 as determined by both markers (1.06 for MSP1_F3 and 1.04 for MS2), indicating that infections were mostly monoclonal. Fig. 3B shows the *P. vivax* molFOB during the entire year of follow-up. Similarly, new *P1s. vivax* infections were restricted to approximately 20% of the study population, whereas 80% did not contract new infections. Of the individuals who had any type of *P. vivax* infection, the majority had a molFOB = 1 to 2. The maximum number of genetically distinct infections/individual/year was molFOB = 5.

*Factors associated with P. vivax molFOB* - Table III shows the results of the multivariate negative binomial model applied to *P. vivax* molFOB and it was significantly associated with living in a community (p < 0.001). *P. vivax* molFOB was lowest in the Brasileirinho community with an aIRR = 0.47 (0.31-0.71), when compared to Ipiranga. No significant difference in *P. vivax* molFOB was observed between the Ipiranga and Puraquequara communities. Increased *P. vivax* molFOB was significantly associated with reported bed net possession [(aIRR:1.63 (1.24-2.15), p < 0.001)] and previous malaria episodes [(aIRR:3.02 (2.02-4.53), p < 0.001)] [Supplementary data (Table IV)].

**TABLE III:** Factors associated with *Plasmodium vivax* molecular force of infection (molFOB). Adjusted incidence rate ratio (aIRR) was calculated using a negative binomial regression model

Risk factor	*P. vivax*_mol_FOB	
	aHR	p
Community (ref. Ipiranga)		
Brasileirinho	0.47 (0.31-0.71)	< 0.001
Puraquequara	0.98 (0.69-1.39)	
Age group (ref. 1-10)		
10-20	1.15 (0.73-1.82)	
21-60	1.06 (0.71-1.60)	0.420
≥ 61	0.75 (0.43-1.29)	
Employed in agriculture^*a*^	1.22 (0.81-1.83)	0.350
Male	1.20 (0.91-1.58)	0.200
Bed net usage^*b,c*^	1.63 (1.24-2.15)	< 0.001
Travel frequency^*c*^	1.00 (0.92-1.10)	0.950
House treated with IRS^2^	0.88 (0.66-1.16)	0.360
Windows protected by screen^1^	1.04 (0.74-1.45)	0.840
Reported previous malaria	3.02 (2.02-4.53)	< 0.001

*a*: status at enrolment; *b*: as time-changing covariate (average observed at time of outcome); *c*: average bed net usage was defined as the proportion of times a person had answered `yes’ to the question: `Did you sleep under a bed net last night’ during ACD; IRS: indoor residual spraying.

## DISCUSSION

The present study highlights well-known differences in the epidemiology of *P. vivax* and *P. falciparum* and evaluated the incidence of *Plasmodium* spp. and clinical cases of malaria in peri-urban communities in the Brazilian Amazon. It also estimated the *P. vivax* molFOB in low transmission settings in Manaus. Understanding malaria epidemiology through *P. vivax* molFOB may provide a detailed assessment of malaria transmission by measuring individual exposure and its burden. Such information is of paramount importance when measuring intervention efficacy, host susceptibility and transmission patterns in low transmission and pre-elimination settings such as Brazil.

Previous studies have measured molFOB in Papua New Guinean children in observational and randomised clinical trial cohorts, and have related incidence of clinical infection and other factors for this measurement.^19^ ^,^ ^28^ The main factors related to *P. falciparum* molFOB in these earlier studies were seasonality, village of residence and age.^18^ ^,^ ^19^ ^,^ ^28^ As for *P. vivax*, molFOB was strongly associated with incidence of clinical episodes and a high molFOB likely resulted in rapid acquisition of immunity against *P. vivax* in children.^19^

Recent studies in the Amazon region and other malaria endemic areas in the world have shown a high proportion of submicroscopic *P. vivax* infections.^7^ ^,^ ^13^ ^,^ ^29^ Even in seasons with the highest malaria transmission, molFOB was low, although higher than that seen in other seasons, showing that molFOB is influenced by different factors. In the present study, factors associated with *P. vivax* positivity were age (20-60 years), seasonality, use of mosquito nets and IRS. In the Brasileirinho, Ipiranga and Puraquequara communities, the distribution of mosquito nets and IRS is mainly focused on areas where there are many cases of malaria (ascertained by active and passive case detection and registered on the SIVEP-Malaria platform). However, this measure may not have been effective in preventing infection in these areas, or it may not be sufficient to decrease *P. vivax* positivity, since many infections may derive from hypnozoites rather than new infections, as suggested by a study in Papua New Guinea.^18^ In addition, it is necessary to consider potential resistance to insecticides or non-acceptance of the use of mosquito nets by the population. These aspects, however, need to be further studied.

A higher prevalence of malaria infection in the rainy season was also shown in previous studies, thus corroborating what has already been described for other regions of the Amazon.^29^ ^,^ ^30^ The predictors of *P. vivax* clinical disease found in our study were seasonality and, marginally, frequency of travel and use of mosquito nets. Koepfli et al.^19^ also found the seasonality to be a predisposing factor in clinical disease by *P. vivax.* Protective factors against clinical disease, such as being over 60 years old and working in agriculture, may be associated with prolonged exposure to *P. vivax* infection during one’s lifetime. Due to age- and exposure-dependent acquired immunity, clinical presentation of malaria becomes rarer, thus increasing the number of asymptomatic carriers.^19^ ^,^ ^31^

We also observed that prevalence of *P. vivax* and the molFOB in the study area were higher and less affected by seasonality when compared to *P. falciparum*. Whereas prevalence of *P. vivax* and the molFOB peaked in November in the Ipiranga community, transmission indicators remained more stable throughout the entire observational period in the other two communities, with almost no seasonality observed in Brasileirinho.

In contrast, the annual *P. falciparum* prevalence profile was characterised by a very sharp peak in January in the Ipiranga community during which the observed prevalence increased by almost 10-fold, alongside a sharp rise of clinical malaria cases. In fact, nearly all clinical cases observed in the present study occurred during this period and in the Ipiranga community. The outbreak caused by *P. falciparum* subsided by April, and the remainder of the observational period was characterised by very low *P. falciparum* prevalence in all three communities. These observed differences in the seasonality profiles of the two species indicate a more stable transmission of *P. vivax* in contrast to a more unstable transmission of *P. falciparum*.

This study has limitations, since the overall lower *P. vivax* parasite densities are close to the threshold of detection of even molecular diagnostic tests, thus hampering the characterisation of the true incidence of new infections detected by genotyping, as reported elsewhere.^32^ ^,^ ^33^ Therefore, estimation of molFOB can be dominated by clones with higher parasite densities, while low density sub-dominant clones may not be detected, especially if they have arisen from relapses. This is an important technical limitation of this and future work and, therefore, the molFOB reported herein is likely an underestimation of the true force of blood-stage infections. Furthermore, it is possible that more inclusions occurred in specific seasons. Although the deforestation variable is important in malaria epidemiology, it was not evaluated in this study. However, the three communities have similar environmental characteristics, and we consider this is not a major bias in our analysis. The population mobility is not properly addressed and its role in the model can be underestimated, being therefore a limitation of this study.

In conclusion, *P. vivax* infection prevailed in the area and infections were mostly observed as being monoclonal. High proportions of symptomatic and submicroscopic infections were also found. Previous malaria episodes were associated with significantly higher *P. vivax* molFOB, likely indicating that effective radical cure is an important strategy to be addressed in these endemic communities. Asymptomatic and submicroscopic infections pose substantial challenges for *P. vivax* malaria control and hamper accurate surveillance efforts that are needed to pursue elimination, especially in low transmission settings.
