# Supplementary material for: Prevalence and force of Plasmodium vivax blood-stage infection and associated clinical malaria burden in the Brazilian Amazon
Source: Mem Inst Oswaldo Cruz. 2022 Jun 24;117:e210330. doi: 10.1590/0074-02760210330 (PMC9239689; doi:10.1590/0074-02760210330)
Supplement: Supplementary file 1 [file 1678-8060-mioc-117-e210330-s.pdf]

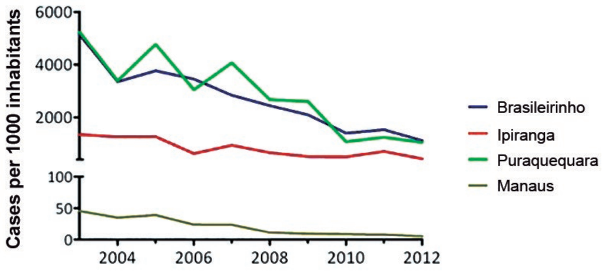

Annual Parasite Index in the study locations. Source: SIVEP Malaria, 2012.

TABLE I

Risk factors associated with *Plasmodium vivax* positivity.
Adjusted hazards ratio (aHR) was calculated using a multiple failure time model

Risk factor	Univariate multiple failure time model		Full multiple failure time model		Back selected multiple failure time model	
	HR	p	aHR	p	aHR	p
Season (Ref: Jun-Nov)	3.34 (1.85-5.75)	< 0.001	2.56 (1.89-5.74)	< 0.001	3.32 (1.9-5.79)	0.00
Community (Ref: Ipiranga)						
Brasileirinho	0.63 (0.42-0.92)	< 0.001	1.01 (0.4-1.08)	0.002	0.63 (0.39-1)	0.01
Puraquequara	1.45 (1.06-1.99)		2.39 (1.11-2.31)		1.5 (1.06-2.12)	
Age group (Ref: < 10)						
10-20	1.13 (0.72-1.78)	0.001	1.36 (0.78-1.95)	0.001	1.21 (0.77-1.9)	0.005
20-60	1.61 (1.15-2.34)		2.22 (1.1-2.45)		1.72 (1.18-2.49)	
60+	1.29 (0.74-2.24)		1.9 (0.71-2.15)		1.34 (0.77-2.31)	
Employed in agriculture	0.78 (0.55-1.10)	0.151	0.82 (0.52-1.12)	0.162	…	…
Male	1.21 (0.91-1.61)	0.192	1.21 (0.91-1.62)	0.187	…	…
Bednet usage[a,c]	1.02 (1.00-1.05)	0.077	1.06 (1.01-1.08)	0.005	1.05 (1.01-1.08)	0.01
Travel frequency[a]	0.99 (0.98-1.00)	0.014	0.99 (0.99-1.01)	0.516	…	…
House treated with IRS[a]	0.97 (0.94-1.01)	0.1	0.96 (0.92-0.99)	0.022	0.96 (0.92-1)	0.03
Windows protected by bug screen[b]	1.07 (0.79-1.47)	0.67	0.78 (0.72-1.37)	0.958	…	…
Reported previous malaria	1.16 (0.71-1.91)	0.55	1.11 (0.56-1.58)	0.814	…	…

a: status at enrolment; *b*: as time-changing covariate (average observed at time of outcome); *c*: average bed net usage was defined as the proportion of times a person had answered `yes' to the question: `Did you sleep under a bed net last night' during ACD; IRS: indoor residual spraying.

CC BY

TABLE II

Risk factors associated with *Plasmodium vivax* clinical disease.
Adjusted hazards ratio (aHR) was calculated using a multiple failure time model

Risk factor	Univariate multiple failure time model		Full multiple failure time model		Back selected multiple failure time model	
	HR	p	aHR	p	aHR	p
Season (Ref: Jun-Nov)	7.57 (2.02-28.33)	0.003	10.56 (2.36-47.19)	0.002	10.64 (2.46-46.04)	0.00
Community (Ref: Ipiranga)						
Brasileirinho	0.14 (0.07-0.29)	< 0.001	0.11 (0.05-0.23)	< 0.001	0.13 (0.06-0.29)	< 0.001
Puraquequara	0.27 (0.16-0.45)		0.26 (0.14-0.5)		0.32 (0.17-0.58)	
Age group (Ref: < 10)						
10-20	0.86 (0.45-1.65)	0.02	1.01 (0.54-1.88)	0.02	0.99 (0.53-1.84)	0.002
20-60	0.89 (0.51-1.53)		0.63 (0.36-1.13)		0.63 (0.35-1.11)	
60+	0.22 (0.05-1.02)		0.15 (0.04-0.63)		0.16 (0.04-0.67)	
Employed in agriculture[b]	0.50 (0.30-0.84)	0.008	0.43 (0.25-0.74)	0.002	0.41 (0.23-0.72)	0.00
Male	1.17 (0.75-1.82)	0.5	1.16 (0.77-1.75)	0.482	…	…
Bednet usage[a,c]	0.97 (0.94-1.01)	0.11	1.06 (1.01-1.11)	0.018	1.06 (1.01-1.11)	0.02
Travel frequency[a]	0.99 (0.97-1.01)	0.46	1.01 (1-1.01)	0.02	1.01 (1-1.01)	0.02
House treated with IRS[a]	0.87 (0.82-0.92)	< 0.001	0.92 (0.86-0.98)	0.008	0.92 (0.86-0.98)	0.01
Windows protected by bug screen[b]	0.55 (0.32-0.95)	0.03	1.56 (0.87-2.8)	0.135	…	…
Reported previous malaria	0.76 (0.19-2.94)	0.65	0.66 (0.17-2.64)	0.558	…	…

a: as time-changing covariate (average observed at time of outcome); *b*: status at enrolment; *c*: average bednet usage was defined as the proportion of times a person had answered `yes’ to the question: `Did you sleep under a bednet last night’ during ACD; IRS: indoor residual spraying.

TABLE III

Risk factors associated with *Plasmodium falciparum* positivity.
Adjusted hazards ratio (aHR) was calculated using a multiple failure time model

Risk factor	Univariate multiple failure time model		Full multiple failure time model		Back selected multiple failure time model	
	HR	p	aHR	p	aHR	p
Season (Ref: Jun-Nov)	0.24 (0.03-2.18)	0.25	0.08 (0.01-0.51)	0.008	…	…
Community (Ref: Ipiranga)						
Brasileirinho	0.05 (0.01-0.21)	< 0.001	0.04 (0.01-0.26)	< 0.001	0.05 (0.01-0.21)	< 0.001
Puraquequara	0.23 (0.10-0.55)		0.23 (0.07-0.71)		0.21 (0.09-0.5)	
Age group (Ref: < 10)						
10-20	0.68 (0.18-2.49)	0.25	0.78 (0.19-3.27)	0.30	…	…
20-60	1.32 (0.42-4.20)		1.33 (0.39-4.5)			
60+	1.33 (0.34-5.14)		2.15 (0.47-9.94)			
Employed in agriculture[b]	0.78 (0.29-1.18)	0.65	1.48 (0.41-5.29)	0.546	…	…
Male	1.16 (0.50-2.69)	0.73	1 (0.4-2.47)	0.996	…	…
Bednet usage[a,c,]	0.91 (0.86-0.97)	0.002	0.95 (0.85-1.06)	0.322	…	…
Travel frequency[a]	0.98 (0.94-1.02)	0.23	1 (0.95-1.05)	0.934	…	…
House treated with IRS[a]	0.91 (0.83- 0.99)	0.03	1.08 (0.94-1.23)	0.278	…	…
Windows protected by bug screen[b]	0.34 (0.10-1.14)	0.08	1.05 (0.27-4.08)	0.946	…	…
Reported previous malaria	6.19 (3.32-11.52)	< 0.001	9.65 (4.45-20.92)	< 0.001	6.32 (3.34-11.96)	< 0.001

a: as time-changing covariate (average observed at time of outcome); *b*: status at enrolment; *c*: average bednet usage was defined as the proportion of times a person had answered `yes' to the question: `Did you sleep under a bednet last night' during ACD; IRS: indoor residual spraying.

TABLE IV

Factors associated with *Plasmodium vivax* molecular force of infection (molFOB).
Adjusted incidence rate ratio (aIRR) was calculated using a Negative Binomial regression model

Risk factor	Univariate negative binomial model		Full negative binomial model		Back selected negative binomial model	
	IRR	p	aIRR	p	IRR	p
Community (ref. Ipiranga)						
Brasileirinho	0.55 (0.38-0.78)	< 0.001	0.47 (0.31-0.71)	< 0.001	0.48 (0.34-0.68)	< 0.001
Puraquequara	1.13 (0.82-1.54)		0.98 (0.69-1.39)		0.92 (0.67-1.25)	
Age group (ref. 1-10)						
10-20	1.4 (0.91-2.18)	< 0.001	1.15 (0.73-1.82)	0.42	…	…
20-60	1.71 (1.19-2.46)		1.06 (0.71-1.6)			
> 60	1.44 (0.86-2.42)		0.75 (0.43-1.29)			
Male	1.21 (0.91-1.59)	0.19	1.20 (0.91-1.58)	0.20	…	…
Travel frequency	0.91 (0.84-0.99)	0.03	1.00 (0.92-1.1)	0.95	…	…
House treated with IRS	1.07 (0.8-1.42)	0.65	0.88 (0.66-1.16)	0.36	…	…
Windows protected by bug screen	1.06 (0.77-1.44)	0.73	1.04 (0.74-1.45)	0.84	…	…
Reported bednet possession	1.56 (1.19-2.05)	0.00	1.63 (1.24-2.15)	< 0.001	1.56 (1.19-2.04)	< 0.001
Reported previous malaria	3.1 (2.16-4.45)	0.00	3.02 (2.02-4.53)	< 0.001	3.06 (2.13-4.39)	< 0.001
Agricultural worker	1.33 (0.92-1.94)	0.14	1.22 (0.81-1.83)	0.35	…	…
